# Beneficial Effects of Nut Consumption on Cognitive Function Among Elderly: Findings From a 6-Year Cohort Study

**DOI:** 10.3389/fnagi.2022.816443

**Published:** 2022-04-13

**Authors:** Fudong Li, Weiping Jiang, Junbiao Wang, Tao Zhang, Xue Gu, Yujia Zhai, Mengna Wu, Le Xu, Junfen Lin

**Affiliations:** ^1^Department of Public Health Surveillance and Advisory, Zhejiang Provincial Center for Disease Control and Prevention, Hangzhou, Zhejiang, China; ^2^Department of Chronic and Noncommunicable Disease Control and Prevention, Haiyan Center for Disease Control and Prevention, Jiaxing, Zhejiang, China

**Keywords:** cognitive impairment, nut consumption, cohort study, elderly, China

## Abstract

**Objective:**

To evaluate the longitudinal association of nut consumption with cognitive function in Chinese elderly.

**Methods:**

We analyzed the data from Zhejiang Ageing and Health Cohort Study including 9,028 participants. Nut consumption was evaluated in baseline questionnaire beginning at 2014. Cognitive function was assessed repeatedly through the Mini-Mental State Examination (MMSE) at baseline and three waves of follow-up (2015, 2016, and 2019–2020). Cognitive impairment was defined using education-specific cut-off points. Log-binomial regression models with the generalized estimating equations, controlled for an extensive range of potential confounders, were utilized to evaluate the association and estimate relative risk (RR).

**Results:**

After 6 years of follow-up, 3,266 (36.18%) participants were indicated as cognitive impairment by MMSE at least once. Compared with non-consumers or less-than-weekly consumers, participants consuming ≥70 g/week of nuts had 17% lower risks of cognitive impairment (RR = 0.83, 95% CI 0.75–0.91), whereas no association was found in those consuming <70 g/week of nuts. Moreover, relatively infrequent higher-amount consuming (≥70 g within one consuming day each week) was not associated with better cognitive performance. Furthermore, we did not observe significant effect modification caused by frequency of other food intake.

**Conclusion:**

Higher nut consumption was prospectively related to a lower risk of cognitive impairment in Chinese elderly.

## Introduction

Cognitive impairment, a common symptom of dementia and its most common cause Alzheimer’s disease (AD), is becoming an important health issue in the elderly (Morley, 2018). It is estimated that more than 35.6 million people lived with dementia worldwide in 2010; this number will be doubled by 2030, and tripled by 2050 (Prince et al., 2013). Based on the estimation, the prevalence of dementia and AD among individuals aged 65 years and older in China were around 5.14% and 3.21%, respectively (Jia et al., 2014), and the situation of cognitive impairment would be more serious.

Accumulating evidence showed that dietary behavior may modify the risk of cognitive impairment and AD (Scarmeas et al., 2018). The inclusion of nuts in the diet has been investigated as a dietary strategy for the maintenance of the brain health across the lifespan (Theodore et al., 2021). Both animal studies and experimental clinical trials demonstrated that nut with its components presented several protective effects for cognitive function, including reduced inflammation, decrease in oxidative stress, beneficial for glucoregulation/insulin sensitivity, ameliorating lipid profile, improvements of endothelial function, cerebral vascular function (Barbour et al., 2014; Alasalvar and Bolling, 2015; Neale et al., 2017), etc.

However, epidemiological data on the association between nut consumption and risk of cognitive impairment among elderly are limited and inconsistent. The majority of cross-sectional studies found a positive relation between nut consumption and cognitive performance (Theodore et al., 2021), whereas causality cannot be inferred due to the nature of study design. In longitudinal studies, some of them supported that nut consumption was protective for cognitive function (Li and Shi, 2019; Rabassa et al., 2020). On the contrary, others observed no association (Nooyens et al., 2011; Samieri et al., 2013). Moreover, the interaction of nut consumption and other factors on risk of cognitive impairment needs to be further explored in cohort studies. In addition, to our knowledge, there is a vacancy in literature clarifying whether the effects of the amount of nut intake on cognitive function vary according to different frequency of nut intake.

Thus in this study, we utilized the data from a large population-based cohort study to investigate the longitudinal association of nut consumption with risk of cognitive impairment among the Chinese elderly.

## Materials and Methods

### Source of Data

**FIGURE 1:**
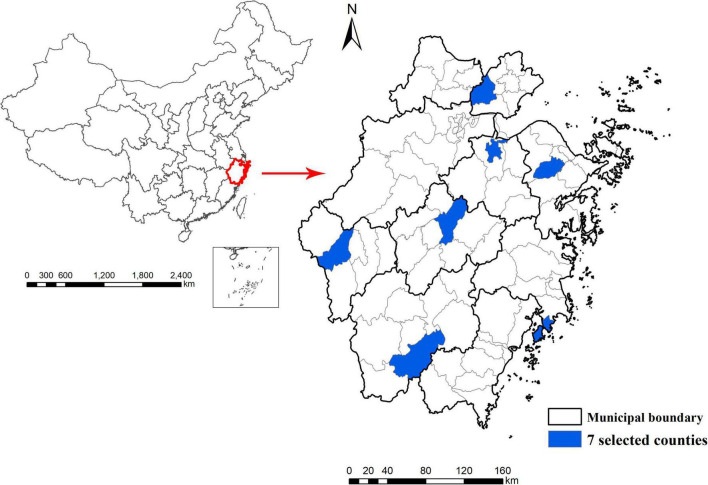
The location of seven selected counties.

The data were from Zhejiang Ageing and Health Cohort Study, a community-based cohort study focusing on aging and health among elderly in Zhejiang province, China. It was conducted by Zhejiang Provincial Center for Disease Control and Prevention. Seven counties were randomly selected from a total of 90 counties in Zhejiang province. The location of seven selected counties is presented in Figure 1. Two counties were urban area, whereas others were rural area. Four counties were above the provincial average according to the gross domestic product (GDP) data from Zhejiang Provincial Bureau of Statistics^1^. One town in each county and several communities in each town were then randomly selected. All permanent residents aged ≥60 years old in these selected communities were eligible and expected to be included in the study.

The baseline survey was initiated in 2014, and 10,901 elderly were recruited and completed the survey (10,911 were primarily documented in database, but 10 records were found duplicated later and were ultimately deleted). Three waves of follow-up survey have been administered in 2015, 2016, and 2019–2020, especially. Due to funding restraint, the second wave of follow-up survey was administered in six counties, whereas other waves of follow-up survey were administered in all seven counties.

A face-to-face interview based on a self-designed questionnaire was performed by trained research assistants for each participant at the baseline survey and each wave of follow-up survey. The questionnaire included the information of demographic characteristics, family status, reproductive history, medical disease, behavioral habits, diet habits, injury, depressive symptoms, self-care ability, and cognitive function. Data were checked by staff at the Zhejiang Provincial Center for Disease Control and Prevention. Missing data and logical errors were fed back to the initial interviewer who would try to complete the dataset by reinvestigating the participants.

The protocol was approved by the Ethics Committee of Zhejiang Provincial Center for Disease Control and Prevention, and written consents or finger prints were obtained from all participants prior to the research.

### Cognitive Assessment

Cognitive function was assessed using the Chinese version of the Mini-Mental State Examination (MMSE; Tombaugh and McIntyre, 1992) in the cognitive function section within the questionnaire of baseline and each follow-up survey, which includes 30 items. The maximum score is 30 points, with higher MMSE scores indicating better cognitive function. The widely accepted cut-off points to define cognitive impairment in China (MMSE Chinese Standard) are education-specific (Katzman et al., 1988): 17/18 for people with lower than primary education level, 20/21 for people with primary education level, and 24/25 for people with higher than primary education level. We defined cognitive impairment as an outcome variable according to this standard.

### Nut Consumption

Nut consumption at baseline was regarded as an exposure variable. In the diet habits section within the baseline questionnaire, two items were related to nut consumption: frequency of nut intake (the number of days consuming nuts per week, days/week) and daily amount of nut intake in the days consuming nuts.

Nut consumption (g/week) was calculated from the frequency of nut intake and daily amount of nut intake in the days consuming nuts. It was categorized into three groups based on previous studies (Barbour et al., 2014; Li and Shi, 2019): none or not weekly, <70 g/week, and ≥70 g/week. Moreover, the frequency of nut intake was recoded into three categories: none or not weekly, 1 day/week, and ≥2 days/week.

### Covariates

Based on findings reported in the literature, variables from the baseline survey described below were considered as potential confounders in our analysis: age (years, continuous variable), gender (“male” and “female”), race (“Han ethnicity” and “minority”), education level (“lower than primary,” “primary,” “junior middle,” “senior middle,” and “college and above”), marital status (“single,” “married,” and “divorced/widowed”), family income (“≤10,000,” “10,001–20,000,” “20,001–50,000,” “50,001–100,000,” and “>100,000” Chinese Yuan/year), body mass index (“<18.5,” “18.5 to <24,” and “≥24” kg/m^2^), smoking (“never,” “past,” and “current”), alcohol drinking (“never,” “past,” and “current”), exercise (“yes” and “no”), tea drinking (“yes” and “no”), hypertension (“presence” and “absence”), diabetes (“presence” and “absence”), coronary heart disease (CHD; “presence” and “absence”), vegetables intake (days/week, continuous variable), fruits intake (days/week, continuous variable), red meat intake (days/week, continuous variable), fish intake (days/week, continuous variable), eggs intake (days/week, continuous variable), and Patient Health Questionnaire-9 (PHQ-9) scale scores (continuous variable). Detailed information on some of these variables are described as follows.

(1) Medical disease section of the questionnaire contained the items on the presence or absence of 16 common diseases, which supposed to be formally diagnosed by physician. Hypertension, diabetes, and CHD were considered in this study. (2) The frequency of other food intake was also collected in the diet habits section of questionnaire. Vegetables, fruits, red meat, fish, and eggs were included in this study. (3) Depressive symptoms were evaluated using PHQ-9 scale (Kroenke et al., 2001), 9-question version of the Primary Care Evaluation of Mental Disorders measured by self-reporting. Total score for the nine items ranges from 0 to 27, with greater values indicating increased severity.

### Statistical Analysis

Descriptive statistics were applied to illustrate the general characteristics of included participants. The associations between general characteristics and nut consumption were examined by ANOVA, Kruskal–Wallis test, or chi-square test as appropriate to variables. We used the log-binomial regression models for repeated measures with the generalized estimating equation (GEE) method (Marschner and Gillett, 2012) to assess the longitudinal effect of nut consumption on risk of cognitive impairment. Crude relative risks (RRs), 95% confidence intervals (CIs), and corresponding *p*-values of cognitive impairment associated with nut consumption were calculated in model 1. In model 2, we adjusted for sociodemographic characteristics including age, gender, race, education level marital status, and family income. In model 3, we additionally adjusted for BMI, hypertension, diabetes, CHD, smoking, alcohol drinking, exercise, tea drinking, vegetables intake, fruits intake, red meat intake, fish intake, eggs intake, and PHQ-9 scale scores. The COPY method (Deddens et al., 2003) was utilized when the log-binomial model failed to converge. Tests for linear trend were simultaneously implemented by assigning the median values to each category of nut consumption and modeling these values as a single continuous variable. We also evaluated whether the association of nut consumption with cognitive impairment differed on frequency of nut intake (1 day/week or ≥2 days/week).

Furthermore, we conducted stratified analyses to test whether observed associations varied by frequency of other food intake. The continuous variables of those factors were transformed into dichotomous variables for stratification. Meanwhile, interactions between nut consumption and these factors were checked through the addition of cross-product terms in the corresponding main-effects models. Finally, sensitivity analyses were adopted to evaluate the robustness of our results, with restriction of participants attending the follow-up survey and completing MMSE at least twice, or using the MMSE score <24 to define cognitive impairment, which is widely accepted in studies among Western population (Tombaugh and McIntyre, 1992).

Statistical analyses were conducted using SAS Software Version 9.4 (SAS Institute Inc., Cary, NC, United States). The *p*-value < 0.05 was considered statistically significant.

## Results

In total, 10,901 participants were enrolled at the baseline survey of Zhejiang Ageing and Health Cohort Study. Participants with cognitive impairment (*n* = 1,467) at baseline and those with incomplete baseline data on cognitive function (*n* = 7) and nut consumption (*n* = 4) were excluded from the dataset. Consequently, 9,423 participants with normal cognitive function at baseline and complete baseline information on nut consumption were treated as a specific cohort in this study.

Among them, 8,257 (87.6%) completed the first wave of follow-up survey. In the second wave of follow-up, 6,298 (79.1%) from six counties completed the survey. In the third wave of follow-up, 7,787 (82.6%) completed the survey. Overall, more than half (5,204, 55.2%) completed all three waves of the follow-up, 2,906 (30.8%) completed two waves of the follow-up, 918 (9.7%) completed only one wave of the follow-up, and 395 (4.2%) never completed any wave of the follow-up. Hence, we included 9,028 (95.8%) who attended the follow-up survey at least once in our analysis.

In the sample of 9,028 participants, 3,266 (36.18%) participants were indicated as cognitive impairment by MMSE at least once during 6 years of follow-up. Table 1 shows the baseline characteristics of included participants according to groups of nut consumption. The mean age of participants was 68.7 years, with a higher mean age in those with lower nut consumption (*p* < 0.01). Participants with higher nut consumption had significantly larger proportions in male, higher education level, married, higher BMI, current smoker, non-drinker, and exerciser. Additionally, other general characteristics were also significantly associated with nut consumption, such as family income, hypertension, CHD, tea drinking, and diets intake (vegetables, fruits, red meat, fish, and eggs). However, nut consumption did not differ by race, diabetes, and PHQ-9 scale scores.

**TABLE 1:** Baseline characteristics of 9,028 included participants according to nut consumption.

Variables	Total (*N* = 9028)	Nut consumption			*p*-value
		None or not weekly (*N* = 7297)	<70 g/week (*N* = 367)	≥70 g/week (*N* = 1364)	
**Age** (years, Mean ± SD)	68.7 ± 7.0	69.0 ± 7.1	67.4 ± 6.5	67.2 ± 6.1	**<0.01**
**Gender** (N, %)					**<0.01**
Male	4441 (49.2)	3527 (48.3)	174 (47.4)	740 (54.3)	
Female	4587 (50.8)	3770 (51.7)	193 (52.6)	624 (45.7)	
**Race** (N, %)					
Han ethnicity	8777 (97.2)	7086 (97.1)	364 (99.2)	1327 (97.3)	0.06
Minority	251 (2.8)	211 (2.9)	3 (0.8)	37 (2.7)	
**Education level** (N, %)					**<0.01**
Lower than primary	4176 (46.3)	3588 (49.2)	127 (34.6)	461 (33.8)	
Primary	4018 (44.5)	3101 (42.5)	206 (56.1)	711 (52.2)	
Junior middle	707 (7.8)	516 (7.1)	27 (7.4)	164 (12.0)	
Senior middle	110 (1.2)	84 (1.2)	6 (1.6)	20 (1.5)	
College and above	14 (0.2)	7 (0.1)	1 (0.3)	6 (0.4)	
**Marital status** (N, %)					**<0.01**
Single	133 (1.5)	113 (1.6)	7 (1.9)	13 (1.0)	
Married	7064 (78.4)	5596 (76.9)	311 (84.7)	1157 (84.9)	
Divorced/widowed	1810 (20.1)	1569 (21.6)	49 (13.4)	192 (14.1)	
**Family income** (N, %)					**<0.01**
≤10,000 CNY/year	2918 (32.4)	2383 (32.7)	100 (27.2)	435 (31.9)	
10,001–20,000 CNY/year	1784 (19.8)	1403 (19.2)	107 (29.2)	274 (20.1)	
20,001–50,000 CNY/year	2302 (25.5)	1768 (24.3)	103 (28.1)	431 (31.6)	
50,001–100,000 CNY/year	1140 (12.6)	933 (12.8)	47 (12.8)	160 (11.7)	
>100,000 CNY/year	874 (9.7)	802 (11.0)	10 (2.7)	62 (4.6)	
**BMI** (N, %)					**<0.01**
<18.5 kg/m^2^	467 (5.2)	393 (5.4)	18 (5.0)	56 (4.1)	
18.5∼ < 24 kg/m^2^	4915 (54.7)	4044 (55.7)	186 (51.4)	685 (50.7)	
≥24 kg/m^2^	3597 (40.1)	2828 (38.9)	158 (43.6)	611 (45.2)	
Hypertension (Presence, N, %)	3966 (43.9)	3242 (44.4)	140 (38.1)	584 (42.8)	**0.04**
Diabetes (Presence, N, %)	810 (9.0)	647 (8.9)	33 (9.0)	130 (9.5)	0.73
CHD (Presence, N, %)	287 (3.2)	213 (2.9)	16 (4.4)	58 (4.3)	**0.02**
**Smoking** (N, %)					**<0.01**
Never	6228 (69.0)	5107 (70.0)	251 (68.4)	870 (63.8)	
Past	1898 (21.0)	1483 (20.3)	89 (24.3)	326 (23.9)	
Current	902 (10.0)	707 (9.7)	27 (7.4)	168 (12.3)	
**Alcohol drinking** (N, %)					**<0.01**
Never	2431 (26.9)	1861 (25.5)	108 (29.4)	462 (33.9)	
Past	726 (8.0)	593 (8.1)	22 (6.0)	111 (8.1)	
Current	5871 (65.0)	4843 (66.4)	237 (64.6)	791 (58.0)	
Exercise (yes, N, %)	1829 (20.3)	1358 (18.6)	87 (23.7)	384 (28.2)	**<0.01**
Tea drinking (yes, N, %)	2282 (25.3)	1749 (24.0)	116 (31.6)	417 (30.6)	**<0.01**
Vegetables intake (d/week, Mean ± SD)	6.5 ± 1.2	6.4 ± 1.3	6.3 ± 1.4	6.7 ± 1.0	**<0.01**
Fruits intake (d/week, Mean ± SD)	2.4 ± 2.2	2.2 ± 2.2	2.7 ± 2.3	3.0 ± 2.3	**<0.01**
Red meat intake (d/week, Mean ± SD)	2.8 ± 2.2	2.8 ± 2.2	3.0 ± 2.0	3.3 ± 2.2	**<0.01**
Fish intake (d/week, Mean ± SD)	2.4 ± 2.3	2.4 ± 2.4	2.0 ± 1.7	2.5 ± 2.3	**<0.01**
Eggs intake (d/week, Mean ± SD)	1.9 ± 2.0	1.8 ± 2.0	1.9 ± 1.6	2.2 ± 2.0	**<0.01**
PHQ-9 scores (Mean ± SD)	1.4 ± 2.4	1.4 ± 2.5	1.3 ± 2.5	1.4 ± 2.3	0.20

*Data for the following are missing: 3 for education level, 21 for marital status, 10 for family income, 49 for BMI, 2 for fruit intake, 4 for fish intake, 1 for eggs intake. BMI, body mass index; CHD, coronary heart disease; CNY, Chinese Yuan; d/week, days per week; g/week, grams per week; SD, standard deviation; PHQ-9, patient health questionnaire-9 scale.*

*The bold values mean “P < 0.05”.*

We initially explored the association between nut consumption and risk of cognitive impairment, and the results are presented in Table 2. Compared with non-consumers or less-than-weekly consumers, participants consuming both <70 g/week and ≥70 g/week of nuts had significant lower risks of cognitive impairment in model 1. After adjustment for sociodemographic characteristics and other covariates, the association was not significant among participants consuming nuts less than 70 g/week (*p* = 0.10). The fully adjusted RR was 0.83 (95% CI: 0.75–0.91) for participants consuming ≥70 g of nut weekly (*p* < 0.01).

**TABLE 2:** Longitudinal association between nut consumption and risk of cognitive impairment.

Nut consumption	Total	Cases	Model 1^a^		Model 2^b^		Model 3^c^	
			RR (95% CI)	*p*-value	RR (95% CI)	*p*-value	RR (95% CI)	*p*-value
None or not weekly	7297	2783	1.00 (Ref)	–	1.00 (Ref)	–	1.00 (Ref)	–
<70 g/week	367	106	0.77 (0.65–0.91)	**<0.01**	0.85 (0.71–1.01)	0.06	0.86 (0.72–1.03)	0.10
≥70 g/week	1364	377	0.73 (0.66–0.80)	**<0.01**	0.81 (0.73–0.89)	**<0.01**	0.83 (0.75–0.91)	**<0.01**
*P* for trend			**<0.01**		**<0.01**		**<0.01**	

*RR, relative risk; CI, confidence interval; g/week, grams per week.*

*^a^No variable was adjusted in model 1.*

*^b^Adjusted for age, gender, race, education level marital status, family income.*

*^c^Adjusted for same covariates in model 2, plus body mass index, hypertension, diabetes, coronary heart disease, smoking, alcohol drinking, exercise, tea drinking, vegetables intake, fruits intake, red meat intake, fish intake, eggs intake, Patient Health Questionnaire-9 scale scores.*

*The bold values mean “P < 0.05”.*

We further investigated whether the observed associations varied according to different frequency of nut intake. For participants consuming nuts once per week, nut consumption was not associated with cognitive function referred to non-consumers or less-than-weekly consumers, regardless of the different amount of nut intake. Among those consuming nuts at least 2 days/week, high nut consumption (≥70 g/week) yielded lower likelihood of cognitive impairment, whereas no identifiable association was found for low-to-moderate nut consumption (<70 g/week) (Table 3).

**TABLE 3:** Adjusted RR (95% CI) for cognitive impairment according to amount and frequency of nut consumption.

Amount and frequency of nut consumption	Total	Cases	RR (95% CI)^a^	*p*-value^a^
**None or not weekly**	7297	2783	1.00 (Ref)	–
**1 day/week**				
<70 g/week	309	90	0.84 (0.69–1.01)	0.06
≥70 g/week	301	90	0.83 (0.68–1.01)	0.06
**≥2 days/week**				
<70 g/week	58	16	1.02 (0.65–1.61)	0.92
≥70 g/week	1063	287	0.81 (0.72–0.90)	**<0.01**

*RR, relative risk; CI, confidence interval; g/week, grams per week.*

*^a^Adjusted for all same covariates included in model 3 (see Table 2).*

*The bold values mean “P < 0.05”.*

To illustrate the potential effect modification caused by frequency of other food intake, stratified analyses were implemented, and the results are demonstrated in Table 4. No obvious difference was found across the subgroups for vegetables, fruits, red meat, fish, and eggs intake. For the subgroup with vegetables intake less than 5 days/week, neither <70 g/week nor ≥70 g/week of nut consumption was associated with risk of cognitive impairment.

**TABLE 4:** Adjusted RR (95% CI) for cognitive impairment with nut consumption, stratified by frequency of other food intake.

Subgroups	Total	Cases	Nut consumption		
			None or not weekly	<70 g/week	≥70 g/week
**Vegetables intake**					
<5 days/week	783	315	1.00 (Ref)	0.85 (0.55–1.30)	0.86 (0.58–1.27)
≥5 days/week	8239	2948	1.00 (Ref)	0.87 (0.72–1.06)	0.81 (0.73–0.89)
*P*_Interaction_	0.15				
**Fruits intake**					
<3 days/week	5542	2039	1.00 (Ref)	0.90 (0.72–1.13)	0.86 (0.76–0.98)
≥3 days/week	3480	1224	1.00 (Ref)	0.82 (0.62–1.08)	0.78 (0.67–0.90)
*P*_Interaction_	0.35				
**Red meat intake**					
<3 days/week	4543	1737	1.00 (Ref)	0.81 (0.63–1.05)	0.87 (0.76–1.01)
≥3 days/week	4479	1526	1.00 (Ref)	0.89 (0.70–1.13)	0.77 (0.68–0.88)
*P*_Interaction_	0.66				
**Fish intake**					
<3 days/week	5565	2099	1.00 (Ref)	0.82 (0.66–1.00)	0.80 (0.70–0.91)
≥3 days/week	3457	1164	1.00 (Ref)	1.02 (0.75–1.40)	0.85 (0.72–0.99)
*P*_Interaction_	0.26				
**Eggs intake**					
<2 days/week	4648	1702	1.00 (Ref)	0.82 (0.64–1.07)	0.86 (0.75–0.99)
≥2 days/week	4374	1561	1.00 (Ref)	0.85 (0.68–1.08)	0.78 (0.68–0.89)
*P*_Interaction_	0.34				

*RR, relative risk; CI, confidence interval; g/week, grams per week.*

*Adjusted for all same covariates included in model 3 (see Table 2), except the variable for stratification.*

The sensitivity analyses, either restricting participants attending the follow-up survey and completing MMSE at least twice, or using 24 as cut-off point for definition of cognitive impairment, yielded similar results to the main analysis (Table 5).

**TABLE 5:** Sensitivity analyses for association between nut consumption and risk of cognitive impairment.

Nut consumption	Total	Cases	Model 1		Model 2		Model 3	
			RR (95% CI)	*P* value	RR (95% CI)	*P* value	RR (95% CI)	*P* value
**Sensitivity analysis 1: in participants attending the follow-up survey and completing MMSE at least twice**								
None or not weekly	6383	2479	1.00 (Ref)	–	1.00 (Ref)	–	1.00 (Ref)	–
<70 g/week	311	90	0.75 (0.62–0.90)	**<0.01**	0.83 (0.69–1.00)	0.05	0.85 (0.70–1.03)	0.09
≥70 g/week	1166	335	0.73 (0.66–0.81)	**<0.01**	0.81 (0.73–0.90)	**<0.01**	0.83 (0.75–0.92)	**<0.01**
*P* for trend			**<0.01**		**<0.01**		**<0.01**	
**Sensitivity analysis 2: using MMSE score <24 to define cognitive impairment**								
None or not weekly	5584	3278	1.00 (Ref)	–	1.00 (Ref)	–	1.00 (Ref)	–
<70 g/week	318	164	0.83 (0.74–0.94)	**<0.01**	0.89 (0.79–1.00)	0.06	0.93 (0.82–1.04)	0.20
≥70 g/week	1158	546	0.79 (0.73–0.85)	**<0.01**	0.90 (0.85–0.97)	**<0.01**	0.93 (0.86–0.99)	**0.03**
*P* for trend			**<0.01**		**<0.01**		**0.02**	

*RR, relative risk; CI, confidence interval; g/week, grams per week; MMSE, mini-mental state examination. Variables in model 1, model 2, and model 3 were same as those in Table 2.*

*The bold values mean “P < 0.05”.*

## Discussion

In this cohort study, we found that higher nut consumption was prospectively related to a lower risk of cognitive impairment among Chinese elderly, which was indicated robust by sensitivity analyses. This association was not significant among participants consuming nuts only once per week. We did not observe significant effect modification caused by frequency of other food intake.

Our current findings are partially consistent with the previous prospective studies. One cohort study (Rabassa et al., 2020), investigating a total of 119 community-dwelling Italians (≥65 years) over a 3-year follow-up, suggested that regular nut consumption was associated with positive change in the MMSE score and lower odds of cognitive decline. Unfortunately, the sample size and follow-up period were limited, and dose–response relationship was not analyzed. Another longitudinal analysis (Li and Shi, 2019), based on data from the China Health and Nutrition Survey (CHNS) involving 4,822 adults aged ≥55 years, demonstrated that nut intake of ≥10 g/day was associated with better cognitive function, consistent with the results of our study. However, the global cognition, assessed by the Telephone Interview for Cognitive Status (TICS) in this study, was relied on auditory and verbal processing skills, lack of other measures such as speed of processing or visual processing. Likewise, a recent prospective research (Jiang et al., 2021) from the Singapore Chinese Health Study indicated that formerly higher intake of nuts was related to lower odds of cognitive impairment in late life. For the latter two studies (Li and Shi, 2019; Jiang et al., 2021), it is noteworthy that no cognitive assessment was conducted at baseline when collecting nut consumption. As a result, they were unable to exclude participants of poor cognitive function at baseline or adjust baseline scores of cognitive assessment. Some cases of cognitive impairment might already exist at baseline, which could fake the temporal relationship. Additionally, other three cohort studies (Nooyens et al., 2011; Samieri et al., 2013; O’Brien et al., 2014) found that nut consumption was not associated with cognitive decline over time, though two of them (Nooyens et al., 2011; O’Brien et al., 2014) demonstrated protective effects in cross-sectional analysis. Some possible reasons for the inconsistency might be the background of dietary intakes in different populations, the average age of participants, and the duration of study follow-up.

In contrast with previous prospective studies, our study complied with the principle of classic cohort study design (Woodward, 2000), by evaluating cognitive function and excluding prevalent cases of cognitive impairment at baseline. Moreover, the associations were estimated using log-binomial models with GEE method to calculate RRs in our study, which were never reported in previous observational studies on this topic. RRs are generally considered preferable to odds ratios (ORs) in prospective studies (McNutt et al., 2003; Marschner and Gillett, 2012). Therefore, our study might more accurately and appropriately describe the associations. In addition, the evidence linking consuming patterns of nuts to the risk of cognitive impairment has been sparse. Our study found relatively that infrequent higher-amount consuming (≥70 g within one consuming day each week) was not associated with better cognitive performance. Instead, more frequent consuming and adequate amount (≥2 days/week, and ≥70 g/week) tended to be beneficial.

Potential mechanism responsible for the association can be chiefly attributed to biological active components of nuts. The high content of unsaturated fatty acids in nuts can exert neuroprotective effect by influencing insulin sensitivity (Riserus, 2008) and reducing inflammation (Ren and Chung, 2007), which are involved in the development of cognitive decline (Barbour et al., 2014). Meanwhile, unsaturated fatty acids are integral components of the neuronal membranes and regulate several processes within the brain, beneficial for preventing AD (Bazinet and Laye, 2014). Antioxidants, in the form of nutrient (e.g., vitamin E and mineral Se) and non-nutrient (e.g., polyphenols) presented in nuts, can positively affect human cognitive function (Collie and Morley, 2007; Alasalvar and Bolling, 2015; Scarmeas et al., 2018), whereas oxidative stress and damage is recognized as a contributor to the occurrence of cognitive impairment (Barbour et al., 2014). Fiber and L-arginine contained in nuts has been shown to improve endothelial function (Lekakis et al., 2002; Brock et al., 2006), which is essential for blood-flow regulation in the brain and cognitive performance (Poels et al., 2008). Furthermore, potassium (K) and magnesium (Mg), naturally rich in nuts, are supported with effects on blood pressure control (Samadian et al., 2016), and hypertension is one of risk factors for cognitive decline and AD. Besides, *in vitro* and experimental studies confirmed an active role of Mg in degenerative neurological disorders through several important pathways (Veronese et al., 2016).

The significant association of higher nut consumption with cognitive function disappeared among participants with lower frequency of vegetables intake, and the reason remained unclear. Vegetables own a high content of antioxidants, folate, lutein, vitamins, and other bioactive substances that can protect against neurodegeneration (Ye et al., 2013; Morris et al., 2018). A systematic review and meta-analysis of observational studies (Mottaghi et al., 2018) concluded that vegetables intake was associated with the reduced risk of cognitive impairment, especially among Chinese population. It is likely that frequent intake of vegetables is fundamentally important for cognitive function, and the protective effect of nut consumption may be weaken when the vegetables intake is insufficient, an issue worth further study.

There are several methodological issues and limitations in this study. First, the amount of nut intake was only inquired at baseline, so the changes in nut consumption were not captured and its effect on cognitive function could not be evaluated. Second, nut consumption was obtained from participants’ subjective recall; therefore, potential misclassification cannot be excluded. This misclassification is probably non-differential, resulting the associations to attenuate toward the null. Third, the association observed in our study cannot be ascribed to any specific component in nut because we did not consider any biomarker of nut intake. Fourth, no information on types of nut intake was collected within questionnaire. As a consequence, it is impossible to determine differences in efficacy between different types of nuts. Fifth, the classification of nut consumption in this study was referred to previous literature (Barbour et al., 2014; Li and Shi, 2019). However, some other studies (Samieri et al., 2013; O’Brien et al., 2014; Jiang et al., 2021) used a broader categories involving amount for less-than-weekly consumers. Unfortunately, we were unable to evaluate the effect of lower nut consumption due to the limitation of questionnaire. Sixth, although many covariates were adjusted in the models, we could not exclude the possibility for existence of residual confounding caused by other unmeasured factors, which might bias our results. Seventh, a number of participants did not complete all three waves, who tended to be older, lower education level, higher proportion in divorced/widowed group, and lower family income. So the bias could not be excluded. Lastly, we did not take any genetic factor into consideration, such as apolipoprotein E (APOE) carrier status.

Despite those limitations, our study has a number of strengths as follows: (1) longitudinal study design, restricting participants of normal cognitive function at baseline, and relatively long duration of follow-up allowed us to assess the temporal relationship between former exposure and subsequent outcome; (2) our study was based on a large community-based sample of the elderly, and the results were relatively robust and might be potentially correlated with a broader population of Chinese elderly; and (3) a wide range of potential confounders was taken into account in our analyses, including sociodemographic characteristics, health condition, behavioral lifestyle, and dietary habits.

## Conclusion

In summary, our study suggests that higher nut consumption may help to reduce the risk of cognitive impairment based on a community-based cohort in Chinese elderly. The addition of weekly consumption of nuts to one’s diet can be a simple way to contribute to the brain health.

## Data Availability Statement

The datasets presented in this article are not readily available due to data regulations and for ethical reasons. Requests to access the datasets should be directed to the corresponding author.

## Ethics Statement

The studies involving human participants were reviewed and approved by the Ethics Committee of Zhejiang Provincial Center for Disease Control and Prevention. The participants provided their written informed consent to participate in this study.

## Author Contributions

FL conducted the study, analyzed the data, interpreted the results, and wrote the manuscript. JL designed the study and critically revised the manuscript. WJ and JW conducted the study and acquired subjects and data. TZ, XG, YZ, MW, and LX conducted the study and interpreted the results. JL supervised the study. All authors contributed to the article and approved the submitted version.

## Conflict of Interest

The authors declare that the research was conducted in the absence of any commercial or financial relationships that could be construed as a potential conflict of interest.

## Publisher’s Note

All claims expressed in this article are solely those of the authors and do not necessarily represent those of their affiliated organizations, or those of the publisher, the editors and the reviewers. Any product that may be evaluated in this article, or claim that may be made by its manufacturer, is not guaranteed or endorsed by the publisher.

## Footnotes

1
http://tjj.zj.gov.cn/
